# Phytochemical profiling and various biological activities of *Phlomis tuberosa* L.

**DOI:** 10.1038/s41598-024-80456-5

**Published:** 2025-03-01

**Authors:** Zhanat Azhikhanova, Mehmet Emin Duru, Selcuk Kucukaydin, Harry Kwaku Megbenu, Meltem Tas Kucukaydin, Galiya Baisalova, Minavar Shaimardan, Nurxat Nuraje, Mehmet Ali Özler

**Affiliations:** 1 Department of Chemistry. L.N. Gumilyov, Eurasian National University, Astana, 010000 Kazakhstan; 2 Department of Chemistry, Faculty of Science, Muğla Sıtkı Koçman University, Muğla, 48000 Turkey; 3 Department of Medical Services and Techniques, Köyceğiz Vocational School of Health Services, Muğla Sıtkı Koçman University, Köyceğiz, Muğla, 48800 Turkey; 4 Department of Chemical and Materials Engineering, School of Engineering and Digital Sciences, Nazarbayev University, Astana, Kazakhstan; 5 National Laboratory Astana, Nazarbayev University, Astana, Kazakhstan

**Keywords:** *Phlomis tuberosa*, Phenolic compounds, Essential oil, Vitamin, Antioxidant, Antidiabetic, Anticholinesterase, Enzyme inhibitory activity, Plant sciences, Chemistry

## Abstract

In this study, antioxidant, anti-diabetic, anti-cholinesterase, anti-urease, anti-tyrosinase activities, chemical composition and vitamin content of extracts and essential oils obtained from flower, aerial part and roots of *Phlomis tuberosa*, which grows naturally in the Kazakhstan were investigated. Chlorogenic acid and ferulic acid were detected as major constituents in the methanol extracts of *P. tuberosa* flower (12.47 and 25.31 µg/g), aerial part (30.95 and 47.82 µg/g) and root (9.79 and 32.56 µg/g). The main vitamins in the extracts were vitamins B3, C, and E. The main constituents of the essential oils from flowers and aerial parts were n-octacosane (14.34% and 25.66%) and hexahydro farnesyl acetone (13.89% and 18.75%). Flower methanol extract exhibited the highest antioxidant activity according to β-carotene-linoleic acid, ABTS (2,2′-Azino-bis(3-ethylbenzothiazoline-6-sulfonic acid) diammonium salt) and CUPRAC (Cupric Reducing Antioxidant Capacity) methods with IC_50_ values of 48.35 ± 0.84, 51.93 ± 0.85 and 65.43 ± 0.27 µg/mL, respectively. The roots hexane extract (IC_50_:103.2 ± 0.99 µg/mL) showed greater α-glucosidase inhibition than acarbose (IC_50_:128.5 ± 0.62 µg/mL). The anti-urease effect of both essential oils were higher compared to all the extracts, and the essential oil of the flowers demonstrated significant butyrylcholinesterase (BChE) inhibitory activity. This study contributes to the traditional therapeutic uses of *P. tuberosa* and emphasizes its value in the development of new therapeutic agents exhibiting antioxidant and anti-diabetic activity.

## Introduction

From ancient times to the present day, plants have been used in nutrition as food and in the treatment of various diseases due to their phytochemical content, beyond the general photosynthetic benefits provided by living organisms. As scientists’ research on plants increases, the bioactive components and medicinal effects of plants are revealed. Thus, seeing the miraculous life in nature through its association, and every day new discoveries showing the fascinating effects of plants, further increases the interest in research on phytochemistry. Medicinal plants constitute a very important source for the discovery and development of new drug molecules due to their rich chemical content and structural diversity of their metabolites^1^. Approximately 40% of conventional (FDA-approved) medicines prescribed today are known to be of natural origin^2^.

*Phlomis*is a member of the Lamiaceae family and has more than 100 species naturally distributed in North Africa, Europe and Asia^3^. There are 13 species of the genus *Phlomis* in Kazakhstan, one of which is *P. tuberosa*L^4^. *P. tuberosa*, which is naturally distributed in Kazakhstan, is used as a traditional folk remedy in Kazakhstan and other Asian countries for the treatment of poisoning, digestive system diseases, tuberculosis, pulmonary and cardiovascular diseases and rheumatoid arthritis and to protect liver function^5,6^. In addition, *P. tuberosa *is used in Tibetan medicine to treat lung and throat diseases and various chronic conditions^7^. To date, *Phlomis *species have been reported to be rich in flavonoids, iridoids and essential oils^5,7–9^. Previous phytochemical studies on *P. tuberosa *have revealed that it contains flavonoids, alkaloids, iridoids and limited research has been conducted on the constituents of its essential oils^4,5,7,10,11^. In the studies conducted so far, mono- and sesquiterpenoids such as α-pinene, limonene, linalool, germacrene D and β-caryophyllene as well as aliphatic hydrocarbons such as essential fatty acids, phytol, hexahydro farnesyl acetone and squalene have been widely identified in the essential oils of *Phlomis *species^4,8,12^. In vitro antioxidant activities have been investigated on extracts or isolated compounds obtained from *Phlomis* species and methanol extracts of *P. fruticosa* and *P. lanata *have been reported to have antioxidant activity^3,13^. Forsythoside B, verbascoside, phenylethyl alcohol glycosides isolated from the methanol extract of the above-ground parts of *P. caucasica*, exhibited strong free radical scavenging activity^14^. It is understood from the literature that the reducing power capacity, iron chelating effect, DPPH (2,2-Diphenyl-1-picrylhydrazyl) free radical, superoxide anion and ABTS cation radical scavenging effects of extracts obtained from *P. herba-venti*, *P. nissolii*, *P. tuberosa *were investigated^3^. The antidiabetic activity potential of *P. aurea* and *P. ocymifolia* has been demonstrated in scientific research reports. In addition, a recent study reported that *P. tuberosa *ethyl acetate fraction showed significant α-glucosidase inhibitory activity^6^. In a study by Kondeva-Burdina et al. (2023), the data obtained from the in vitro study on the effects of *P. tuberosa* methanol extract with oil removed were confirmed in the in vivo experiment conducted in the CCl_4_-induced hepatotoxicity model in rats, and it was reported that *P. tuberosa *extract with high flavonoid content showed the same effect as silymarin^9^.

Vitamins are essential organic compounds found naturally in plants and fungi and have various physiological functions in the human body. Water-soluble vitamins support metabolism, skin, muscle tone, bone health, immune and nervous systems. In addition, fat-soluble vitamins play important roles in cell functioning, antioxidant defense, blood clotting, immune system support and hormone regulation^15^. Besides a wide range of bioactive compounds such as flavonoids, alkaloids, terpenoids, lipids and essential oils, plants are also an important source of vitamins. The aim of this study was to comprehensively investigate the phenolic constituents, essential oil composition, vitamin content, in vitro antioxidant, antidiabetic, anticholinesterase, urease and tyrosinase enzyme inhibition activities of various parts (flower, aerial part and roots) of *P. tuberosa* L., a unique species native to the Ortau Mountains in Karaganda, the central region of Kazakhstan. Accordingly, acetylcholinesterase, butyrylcholinesterase, urease and tyrosinase enzyme inhibition activities as well as vitamins of *P. tuberosa* were investigated for the first time in this study.

## Materials and methods

### Plant material

**Fig. 1:**
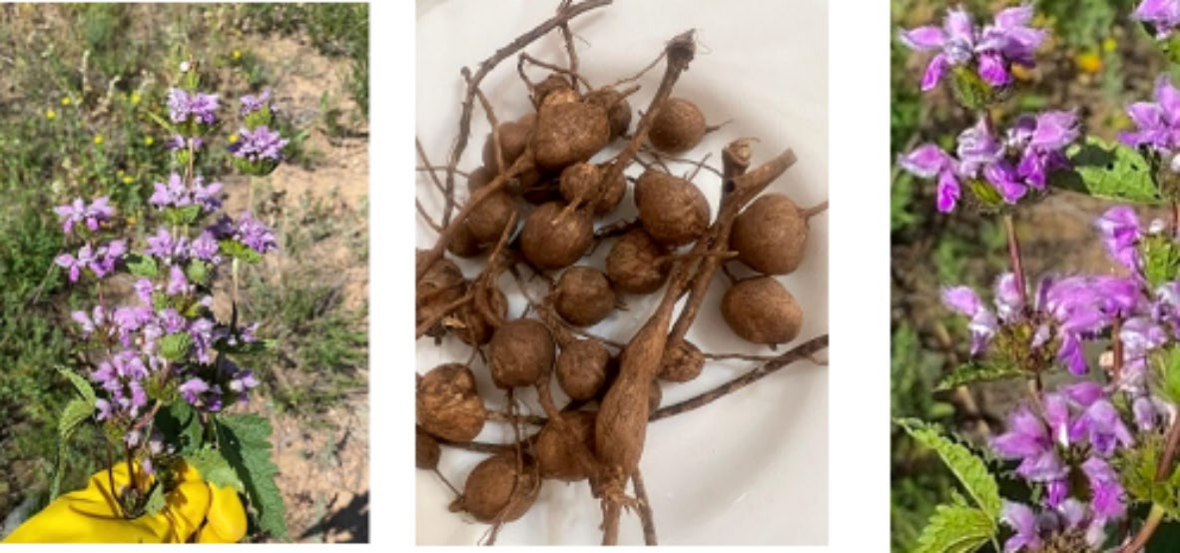
The images of *P. tuberosa*.

*P. tuberosa* L. was collected in June 2021 from the mountains of Ortau near the village Ortau of Karaganda region, Kazakhstan (Fig. 1). The plant was identified by Alibekov D.T., who had his master’s degree in Ecology and is currently a senior researcher at the ‘Laboratory of Flora and Plant Resources’ of the Astana Botanical Garden in Kazakhstan’s capital. The collected plant material was air-dried in the absence of light at room temperature for three weeks. The voucher specimen was then stored at the Natural Products Laboratory, Faculty of Science, Muğla Sıtkı Koçman University, with the voucher number MUP1024.

### Reagents and chemicals

The analytical grade chemicals that were utilized were all purchased from Sigma-Aldrich Chemical Co. (St. Louis, MO, USA) and Merck (Darmstadt, Germany). The analytical grade chemicals that were utilized were all purchased from Sigma-Aldrich Chemical Co. (St. Louis, MO, USA) and Merck (Darmstadt, Germany). These chemicals include, n-hexane, methanol, ethanol, dimethyl sulfoxide (DMSO), β-carotene, butylated hydroxyanisole (BHA), linoleic acid, 2,2′-azino bis (3-ethylbenzothiazoline- 6-sulfonic acid) diammonium salt (ABTS), α-tocopherol, neocuproine, copper (II) chloride, 3-(2-Pyridyl)−5,6-di(2-furyl)−1,2,4-triazine-5′,5′′-disulfonic acid disodium salt (ferene), ethylenediaminetetraacetic acid (EDTA), ferrous chloride, 1,1-diphenyl-2-picryl-hydrazyl (DPPH), galantamine, 5,5′ -dithiobis(2-nitrobenzoic) acid (DTNB), polyoxyethylene sorbitan mono palmitate (Tween-40), sodium bicarbonate (NaHCO_3_), acetylthiocholine iodide, Acetylcholinesterase (AChE) from electric eel (Type-VI-S, EC 3.1.1.7), urease from jack bean (type-III, EC 3.5.1.5), sodium nitroprusside, butyrylthiocholine chloride, phenol, sodium hydroxide, sodium hypochlorite, Butyrylcholinesterase (BChE) from equine serum (EC 3.1.1.8), and urea.

### Extraction

Different parts (flowers, aerial parts, and roots) of *P. tuberosa* L., dried and ground in the shade, were weighed as 50 g each and extracted in an ultrasonic bath at 30 °C (30 min x 3 times) in n-hexane and methanol, respectively. Thus, the solvents (hexane and methanol) of the extracts obtained were removed in the rotary evaporator. All extracts were stored in a refrigerator (+4 °C) until analysis.

### Obtaining of essential oils

Dried different parts of *P. tuberosa *L. were subjected to hydrodistillation using Clevenger apparatus (approximately 100 g for 4 h)^16^. Essential oils thus obtained were dried over anhydrous Na_2_SO_4_ and stored appropriately in dark glass bottles at 4 °C until analysis.

### GC-FID and GC-MS analysis

The chemical compositions of the essential oils were analyzed using GC-FID and GC–MS methods^26^. For GC analysis, a Flame Ionization Detector (FID) and a Rxi-5MS (Restek) fused silica capillary non-polar column (30 m × 0.25 id., film thickness 0.25 μm) were used. The injector temperature and detector temperature were adjusted 250 and 270 °C, respectively. Carrier gas was He at a flow rate of 1.4 mL/ min. Sample size was 0.2 µL with a split ratio of 20:1. The initial oven temperature was held at 60 °C for 5 min, then increased up to 240 °C with 4 °C/min increments and held at this temperature for 10 min. The percentage composition of the essential oil was determined by the Class GC10 GC computer program. In the GC–MS analysis, an ion trap MS spectrometer and a Rxi-5MS (Restek) fused silica non-polar capillary column (30 m×0.25 mm ID, film thickness 0.25 μm) were utilized. Carrier gas was helium at a flow rate of 1.4 mL/min. The oven temperature was held at 60 °C for 5 min, then increased up to 240 °C with 4 °C/min increments and held at this temperature for 10 min. Injector and MS transfer line temperatures were set at 220 °C and 290 °C, respectively. The ion source temperature was 200 °C. The injection volume was 0.2 µL with a split ratio of 1:20. EI–MS measurements were taken at 70 eV ionization energy. Mass range was from m/z 28 to 650 amu. Scan time 0.5 s with 0.1 inter scan delays. Identification of components of the essential oils was based on GC retention indices determined using a homologous series of C7-C30 alkanes (Supelco) and computer matching with the Wiley, NIST14 and TRLIB Library as well as by comparison of the fragmentation patterns of the mass spectra with those reported in the literature^17^ and whenever possible, by co-injection with authentic compounds. Quantification of each essential oils individual constituents was based on internal normalization for the components.

### Determination of vitamins

The vitamin B group and C content in the extracts was measured using reverse-phase high-performance liquid chromatography (HPLC) according to a published standard method with some modifications^18,19^.

Vitamin E content in each extract was measured using reverse-phase HPLC with a fluorometric detector according to the method described in the published standard method with some modifications^18^.

### Determination of phenolic composition

A Shimadzu 20 AT series high-performance liquid chromatograph with a diode array detector (HPLC-DAD) (Shimadzu Cooperation, Kyoto, Japan) was used to analyze the phenolic compounds. The obtained extracts were dissolved in water: methanol (80:20) and filtered through a 0.20 μm disposable LC filter disk to remove suspended particles before loading on an Intersil ODS-3 reverse phase C18 column for separation and detection^20^. The solvent flow rate was 1.0 mL/min, and the injection volume of the sample was 20 µL. The mobile phase system consists of 0.5% acetic acid in water as mobile phase A and mobile phase B was 0.5% acetic acid in methanol. The elution gradient was as follows: 0–10% B (0–0.01 min); 10–20% B (0.01–5 min); 20–30% B (5–15 min); 30–50% B (15–25 min); 50–65% B (25–30 min); 65–75% B (30–40 min); 75–90% B (40–50 min) 90 − 10% B (50–55 min). The detection was carried out at 280 nm wavelength. The phenolic compounds were characterized by comparing UV data and retention times with commercial standards. The phenolic compounds were quantified using calibration curve established *via* the injection of known concentrations of standard compounds. Total 26 standard phenolic compounds were used namely, gallic protocatechuic, chlorogenic, *p*-hydroxy benzoic, caffeic, 3-hydroxy benzoic, syringic, *p*-coumaric, ferulic, ellagic, rosmarinic, trans-cinnamic acids, catechin, pyrocatechol, 6,7-dihydroxy coumarin, vanillin, taxifolin, coumarin, rutin, myricetin, quercetin, luteolin, hesperetin, kaempferol, apigenin and chrysin. The results were given in (µg g^−1^) of dry weight.

### Determination of antioxidant activity

Antioxidant activities of all extracts were assessed by using different methods including β-carotene-linoleic acid assay, DPPH assay, ABTS assay, CUPRAC assay and Metal chelating assay. Inhibition of lipid peroxidation activity was performed by using β-carotene-linoleic acid test system according to the standards protocol developed by (Marco, 1968) with minor modifications^21,22^. The DPPH assay was performed by using a spectrophotometer according to typical methods documented previously^23^. The ABTS^+^assay was carried out as described previously by Re et al., (1999)^24^. The cupric reducing antioxidant capacity (CUPRAC) was evaluated by following the method published Apak et al., (2004)^25^. α-tocopherol and butylated hydroxy anisole (BHA) were used as antioxidant standards to compare the β-carotene-linoleic acid, DPPH, ABTS and CUPRAC assays. The metal chelating assay of extracts for Fe^+2 ^was performed by using a spectrophotometer as the method reported by Tel et al., (2012)^26^. Ethylenediaminetetraacetic acid (EDTA) was used as a standard. Antioxidant activity results were expressed as IC_50_ (µg/mL) values and percentage inhibitions at 200 µg/mL concentrations.

### Determination of anticholinesterase activity

The inhibitory activities of acetylcholinesterase and butyrylcholinesterase enzymes of the extracts were determined using spectrophotometer following the protocol described by Deveci et al., (2019) with slight modifications^27^. Acetylthiocholine iodide and butyrylthiocholine chloride were utilized as reaction substrates, while AChE from electric eels and BChE from horse serum were applied. Employing DTNB (5,5′-Dithio-bis(2-nitrobenzoic) acid), the cholinesterase activity was measured. In a nutshell, 130 µL of 100 mM sodium phosphate buffer (pH 8.0), 10 µL of sample solution at different concentrations, and 20 µL of enzyme (AChE or BChE) solution in buffer were mixed and incubated for 15 min at 25 °C, and 20 µL of 0.5 mM DTNB was added. Following that, 0.71 mM, 20 µL of acetylthiocholine iodide or 0.2 mM, 20 µL of butyrylthiocholine chloride were added to initiate the reaction. Utilizing a 96-well microplate reader, the hydrolysis of these substrates was observed spectrophotometrically by the formation of a yellow 5-thio-2-nitrobenzoate anion at a wavelength of 412 nm. The Galantamine was used as a reference compound. Results were given as IC_50_ (µg/mL) values and percentage inhibitions at 200 µg/mL concentrations.

### Determination of urease inhibition activity

The inhibitory activity of the urease enzyme by each extract of *P. tuberosa *were evaluated by determining ammonia production with the indophenol method^28^ using a microplate reader. Briefly, 25 µL of enzymatic urease solution (jack bean source), 50 µL Urea (100 mM) and (100 mM) of sodium phosphate buffer (pH 8.2) were mixed and incubated at 30 °C for 15 min after adding the sample (10 µL of extracts). Then 70 µL of alkali reagent and 45 µL of phenol reagent were added to each well. After 50 min of incubation, the absorbance was recorded at 630 nm using a microplate reader. The reference compound was used as thiourea. Urease inhibitions results were presented as IC_50_ (µg/mL) values and percentage inhibitions at 200 µg/mL concentrations.

### Determination of tyrosinase inhibition activity

Tyrosinase enzyme inhibitory activity was measured by the spectrophotometric method as described by Masuda et al., (2005)^29^. L-DOPA was utilized as substrate of the reaction. 150 µL of sodium phosphate buffer (pH 6.8, 100 mM), 10 µL of sample and 20 µL of tyrosinase enzyme solution in buffer were mixed and incubated for 10 min at 37 °C. Following incubation, 20 µL of L-DOPA was added. The absorbances in a 96-well microplate were monitored at 475 nm after 10 min of incubation at 37 °C. Kojic acid was used as a reference compound. Results were provided as percentage inhibitions at 200 µg/mL and IC_50_ (µg/mL) values.

### Determination of antidiabetic activity

The α-amylase inhibitory activity was evaluated by using starch-iodine method^30^. The enzyme α-amylase from porcine pancreas was used and enzyme solution was prepared with phosphate buffer (20 mM pH = 6.9 phosphate buffer prepared with 6 mM NaCl). Then, 50 µL of α-amylase and 25 µL of sample solutions were mixed in a 96-well microplate. The mixture was pre-incubated for 10 min at 37 °C. Then, 50 µL of starch solution (0.05%) was added and incubated for 10 min at 37 °C. Following incubation, the reaction was completed by adding HCl (0.1 M, 25 µL) and Lugol (100 µL) solutions, and the absorbance was recorded at 565 nm.

The α-glucosidase inhibitory activity was evaluated according to the method described previously^31^. 50 µL of phosphate buffer (0.01 M pH 6.9), 10 µL of sample solution, 50 µL of α-glucosidase from *Saccharomyces cerevisiae* in phosphate buffer (0.01 M pH 6.0) and 25 µL of PNPG (4-N-nitrophenyl-α-d-glucopyranoside) in phosphate buffer (0.01 M pH 6.9) were mixed in a 96-well microplate. Then the solution was incubated for 20 min at 37 °C. Acarbose was used as standard compound for both analyses. Antidiabetic activity results were given as IC_50_ (µg/mL) values and percentage inhibitions at 200 µg/mL concentrations.

### IC_50_ and A_0.50_ values

IC_50_ (50% inhibition concentration) or A_0.50_ (absorbance of 0.50) values were calculated whenever possible to express the results. IC_50_ values were calculated using a graph plotted between concentration (µg/mL) and percentage inhibition (%). A graph that plotted absorbance against concentration (µg/mL) was used to determine A_0.50_ values.

### Statistical analysis

All data were analyzed in triplicate. Data were expressed as means ± standard deviation of three samples. Statistical analysis was performed with MINITAB 16. Differences were tested for significance by using the ANOVA (analysis of variance) procedure with a significance level of *p* < 0.05.

## Results

### Chemical composition of essential oils

**Fig. 2:**
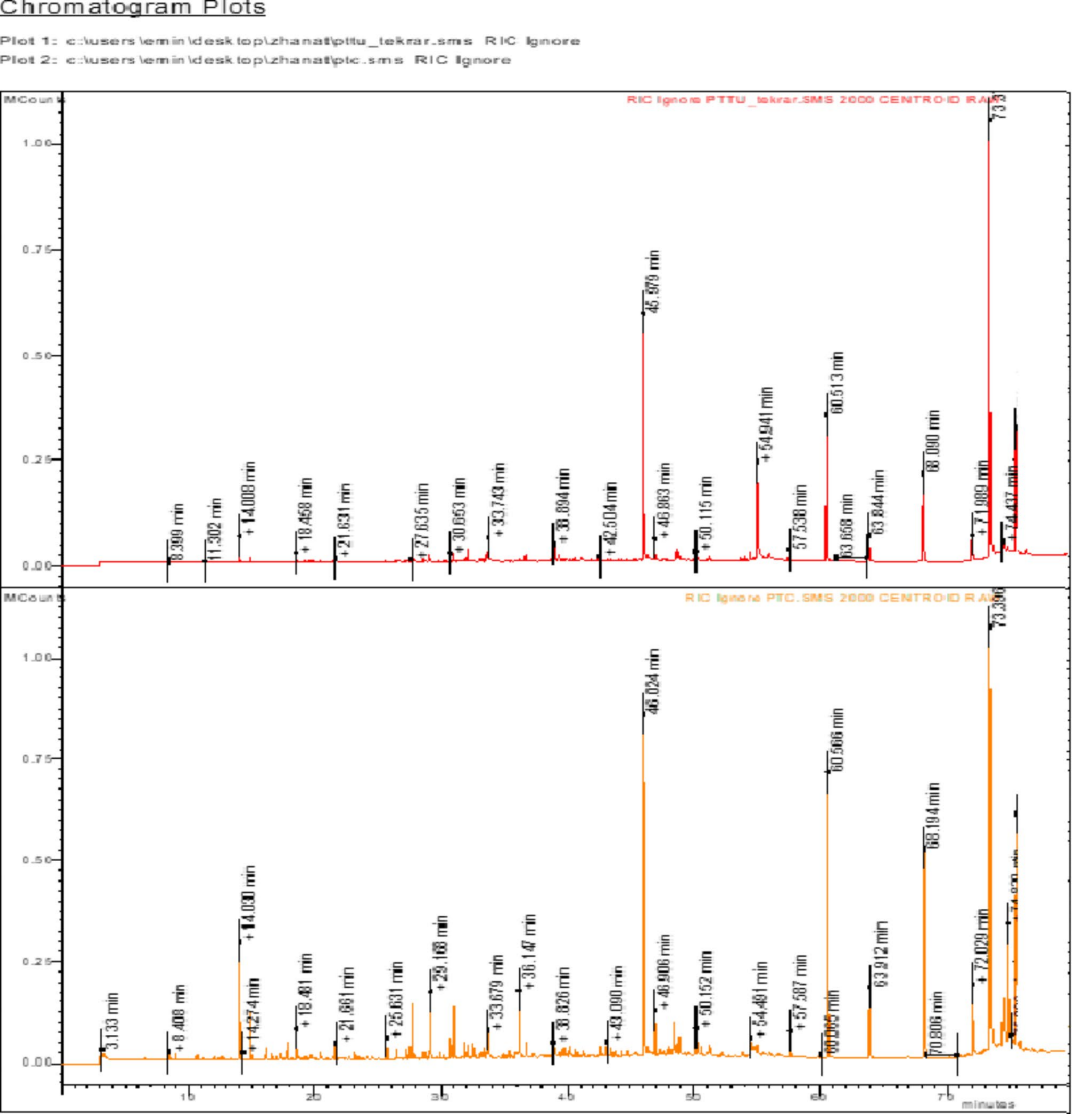
GC-MS Chromatograms of essential oils of aerial parts and flowers from *P. tuberosa*.

Essential oils of the flowers and aerial parts of *P. tuberosa* gave a yellowish oil with a yield of 0.62% and 0.73% (w/w), respectively. Both essential oils are in liquid form, light yellow in color, and have a distinctive, pungent odor. To determine the components of the obtained essential oils qualitatively and quantitatively, they were analyzed by Gas Chromatography (GC) and Gas chromatography-mass spectrometry (GC-MS). In GC and GC/MS analyses, 49 components detected in the essential oil obtained from *P. tuberosa* flowers and 47 components detected in the aerial parts were determined using NIST library data, reference compounds and RI (retention indices) values (Table 1). GC-MS chromatograms of the essential oil are presented as Fig. 2. According to these analyses, in the essential oil of the flower and aerial parts of *P. tuberosa*; n-octacosane (14.34% and 25.66%), hexahydro farnesyl acetone (13.89% and 18.75%), n-pentacosane (13.31% and 9.29%), docosane (10.37% and 9.31%), linalool (4.27% and 1.84%), caryophyllene oxide (2.91% and 1.21%) and *β*-Caryophyllene (2.55% and 1.35%) were found to be main compounds. The identified chemical compounds are listed in detail in (Table 1) along with their retention index (RI). When the 51 components obtained in the analysis of the essential oil obtained from *P. tuberosa* flowers and aerial parts were categorized in terms of compound classes, the majority of the oil was aliphatic hydrocarbons (55.50% and 66.12%, respectively), sesquiterpenoids (28.46% and 26.60%) and monoterpenoids (13.02% and 4.55%). Although the essential oil components of flowers and aerial parts do not differ much qualitatively, they show a significant difference quantitatively (Table 1).

**Table 1:** Essential oil composition of the flowers and aerial parts of *P. Tuberosa* L.

No	Compound	RI^a^	LRI^b^	Flowers (%)^c^	Aerial parts (%)^c^	Identification methods^d^
1	1-Octen-3-ol	962	961	0.28	0.15	Co-GC, MS, RI
2	2-Pentyl furan	977	974	0.19	-	MS, RI
3	Limonene	1016	1015	0.16	-	Co-GC, MS, RI
4	1,8-Cineole	1022	1025	0.16	-	Co-GC, MS, RI
5	Phenylacetaldehyde	1026	1028	-	0.07	Co-GC, MS, RI
6	cis-Linalool oxide	1060	1061	0.34	-	Co-GC, MS, RI
7	Linalool	1083	1083	4.27	1.84	Co-GC, MS, RI
8	2-Nonen-1-ol	1091	1092	0.29	0.05	Co-GC, MS, RI
9	2.5-Diethyl thiophene	1097	1100	1.40	0.24	MS, RI
10	Camphor	1118	1120	0.44	*tr*	MS, RI
11	Lilac aldehyde B	1143	1145	0.20	0.05	Co-GC, MS, RI
12	[E]−2-nonenal	1149	1152	0.09	-	MS, RI
13	Borneol	1155	1160	0.38	0.23	Co-GC, MS, RI
14	Terpinen-4-ol	1159	1163	0.62	tr	Co-GC, MS, RI
15	α-Terpineol	1175	1177	1.72	0.95	Co-GC, MS, RI
16	2.6-Dimethyl-4-thiopyrone	1178	1181	0.16	0.09	MS, RI
17	p-Menth-1-en-9-al	1183	1185	0.12	0.1	MS, RI
18	cis-Geraniol	1208	1211	0.55	0.23	Co-GC, MS, RI
19	Methyl 3-(butylsulfanyl)propanoate	1235	1239	0.12	0.09	MS, RI
20	trans-Geraniol	1242	1246	0.99	0.64	MS, RI
21	Bornyl acetate	1251	1253	0.29	-	Co-GC, MS, RI
22	Dihydroedulan IA	1292	1297	0.20	0.14	MS, RI
23	3.3.4-Trimethyl-1.3-dihydro-pyrrole-2-thione	1323	1326	0.74	0.15	MS, RI
24	Eugenol	1327	1332	0.33	0.17	Co-GC, MS, RI
25	α-Copaene	1352	1355	0.36	-	MS, RI
26	α -Gurjunene	1361	1368	2.34	0.79	MS, RI
27	β-Cubebene	1394	1400	0.56	0.24	Co-GC, MS, RI
28	β-Caryophyllene	1414	1415	2.55	1.35	Co-GC, MS, RI
29	Dihydropseudoionone	1427	1434	0.89	0.67	MS, RI
30	α-Selinene	1478	1483	0.92	0.34	MS, RI
31	Myristicin	1523	1530	1.59	0.89	MS, RI
32	Caryophyllene oxide	1573	1575	2.91	1.21	Co-GC, MS, RI
33	β-Eudesmol	1620	1627	0.66	0.21	MS, RI
34	Agaruspirol	1631	1635	0.60	1.35	MS, RI
35	α-Methyl ionone	1639	1642	0.25	0.49	MS, RI
36	α-Bisabolol	1650	1655	0.45	0.14	Co-GC, MS, RI
37	cis-10-Pentadecen-1-ol	1692	1698	0.38	0.14	MS, RI
38	α-Hexylcinnamaldehyde	1726	1735	0.31	0.32	MS, RI
39	Cembrene	1824	1827	0.49	0.17	MS, RI
40	Hexahydro farnesyl acetone	1833	1836	13.89	18.75	MS, RI
41	Diisobutyl phthalate	1863	1869	1.69	1.77	MS, RI
42	cis-9-Eicosen-1-ol	1867	1875	0.38	12.86	MS, RI
43	9-Hexadecene	1949	1953	-	1.47	MS, RI
44	n-Heneicosane	2108	2110	0.98	0.73	MS, RI
45	n-Docosane	2209	1212	10.37	9.31	MS, RI
46	n-Tetracosane	2407	2412	3.46	2.34	MS, RI
47	n-Pentacosane	2508	2511	13.31	9.29	MS, RI
48	n-Heptacosane	2705	2712	3.27	2.22	MS, RI
49	n-Octacosane	2804	2812	14.34	25.66	MS, RI
50	Squalene	2818	2820	6.31	0.87	MS, RI
51	n-Nonacosane	2904	2907	2.70	1.23	MS, RI
Monoterpenoids				13.02	4.95	
Sesquiterpenoids				28.46	26.60	
Aliphatic compounds				55.50	66.12	
Others				3.02	2.33	
Total				100.00	100.00	

^a^Retention index experimentally determined using homologous series of C7-C30 alkanes on Rxi-5Sil MS fused silica column.^b^Literature retention index values, ^c^Percentage concentration, ^d^Identification methods, Co-GC: co-injection based on comparison with standard compounds; MS: based on comparison with WILEY, ADAMS and NIST 14 MS databases.

### Phenolic compounds

**Fig. 3:**
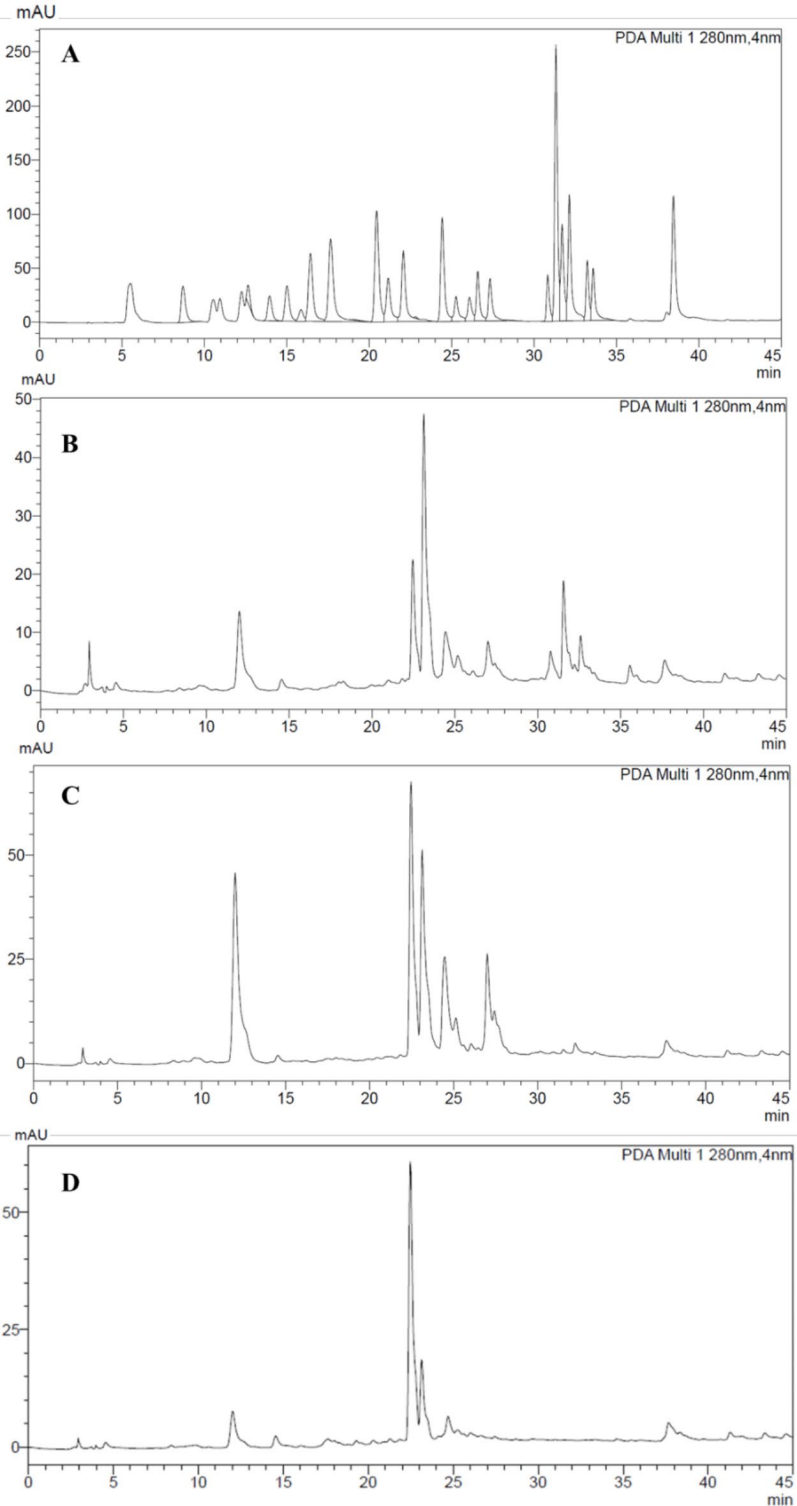
HPLC Chromatograms of reference compounds (**A**), methanol extracts of flower (**B**), aerial parts (**C**) and roots (**D**) from *P. tuberosa*.

Phenolic compound profile of three different parts (flowers, aerial parts and underground) of *P. tuberosa* growing naturally in Kazakhstan was investigated by HPLC-DAD system. The results obtained are given in Table 2 and HPLC chromatograms are given in Fig. 3. A total of 10 phenolic compounds, including chlorogenic acid, ferulic acid, coumarin, rosmarinic acid, quercetin and luteolin, were determined in the flowers, aboveground and underground parts of *P. tuberosa*. Accordingly, protocatechuic acid (1.24, 1.49 and 0.36 µg/g), chlorogenic acid (12.47, 30.95, 9.79 µg/g), ferulic acid (25.31, 47.82 and 32.56 µg/g) and rutin (6.47, 8.34 and 3.95 µg/g) and ellagic acid (2.43, 2.76 and 0.22 µg/g) are common components in all samples (Table 2). Ferulic acid (25.31–47.82 µg/g) and chlorogenic acid (9.79–30.95 µg/g) stand out as common major compounds in all three parts of *P. tuberosa*.

**Table 2:** Phenolic compounds of *P. tuberosa* (µg/g).

No	Phenolic compounds	RT (min)	*P*. tuberosa (flower)	*P*. tuberosa (aerial part)	*P*. tuberosa (underground)
1	Protocatechuic acid	8.75	1.24 ± 0.15	1.49 ± 0.22	0.36 ± 0.04
2	Chlorogenic acid	12.35	12.47 ± 0.77	30.95 ± 0.63	9.79 ± 0.18
3	Ferulic acid	22.14	25.31 ± 0.68	47.82 ± 0.80	32.56 ± 0.45
4	Coumarin	24.49	11.62 ± 0.51	14.91 ± 0.74	nd
5	Rutin	25.30	6.47 ± 0.37	8.34 ± 0.43	3.95 ± 0.12
6	Ellagic acid	26.11	2.43 ± 0.19	2.76 ± 0.11	0.22 ± 0.03
7	Rosmarinic acid	26.77	8.97 ± 0.45	14.51 ± 0.34	nd
8	Quercetin	30.83	5.37 ± 0.36	nd	nd
9	Luteolin	31.70	9.72 ± 0.62	4.40 ± 0.42	nd
10	Kaempferol	33.21	2.58 ± 0.18	2.05 ± 0.23	nd

^a^Values expressed are means ± S.E.M. of three parallel measurements (*p* < 0.05). nd: not detected.

### Vitamins

The composition of vitamins in *P. tuberosa* flowers, and roots was identified and quantified by HPLC analysis where the presence of high concentrations of water-soluble (B and C) and fat-soluble (E) vitamins were determined as shown in Table 3. The content of vitamins obtained from the flowers and the roots varied, and the vitamins were successfully separated from the HPLC analytical instrument. The results obtained from flowers showed the presence of several vitamins ranging from 0.69 mg/100 g to 55.43 mg/100 g, where the highest concentration was observed in vitamin B3 while the lowest concentration was in vitamin B5, as shown in Table 3. Derived from *P. tuberosa* L. roots, substantial quantities of vitamins B1, B2, B3, B5, and B6 were identified, along with a noteworthy vitamin C and E content. The content of all vitamins in all plant materials is expressed in mg/100 g of dry plant material, and the peak results of vitamin content are shown in Table 3. As depicted in Table 3, thiamine (vitamin B1), was obtained from the flowers and roots and possessed small amounts of concentration, such as 2.14 mg/100 g and 1.02 mg/ 100 g, respectively. Thiamine is vital in nerve cell performance, carbohydrate metabolic activities, and energy production, which are crucial in every plant’s life process^32^. Riboflavin (vitamin B2) was measured in the flowers and the roots, where the concentration in the flowers was 1.38 mg/100 g, and that of the roots gave a minute concentration of 0.85 mg/100 g. This implies that the composition of riboflavin is higher in the flowers compared with the amount recorded in the roots. Riboflavin is also an enzyme’s cofactor required for energy metabolism. In the mitochondria respiratory system, riboflavin is an electron carrier, improves skin health, and aids clear vision^32^. Niacin (vitamin B3), a precursor of nicotinamide adenine dinucleotide phosphate and nicotinamide adenine nucleotide, which are respectively proton and electron acceptors, were measured in the flowers and roots. From the experimental result, the amount of vitamin B3 had the highest concentration in the *P. tuberosa *L. flowers (55.43 ± 5.54%). The extraction route could favor the higher concentration of niacin obtained. Meanwhile, from the roots, the amount of niacin detected was 9.99 mg/100 g, which is far lower than the concentration measured in flowers. Pantothenate (vitamin B5) concentration was relatively lower in the flowers (0.69 mg/100 g), while in the roots, the concentration obtained was 1.46 mg/100 g. Pantothenate serves as an acyl protein carrier and a precursor for coenzyme A (CoA)^33^. Pyridoxine (vitamin B6) was also quantified, and the amount detected in flowers (2.74 mg/100 g) was much higher compared with the concentration measured in the roots (0.53 mg/100 g). Pyridoxine improves protein, fatty acid metabolism, and hematopoietic cellular production^34^. In general, the concentration of the B vitamins recorded in *P. tuberosa* L. flowers is much higher than the amount obtained from the roots. This could be attributed to several factors, including nutrient distribution within the plant and the plant’s metabolic processes. Flowers are metabolically active tissues involved during pollination and development, which may require the support of certain nutrients such as the B vitamins in higher concentration in support of biosynthetic demands and energy. On the contrary, roots cater to the nutrient and water absorption from soil while these nutrients are distributed through transport mechanisms to different parts of the plants. An appreciable amount of vitamin C, a water-soluble vitamin, was also detected in flowers 21.02 ± 2.10% and roots 26.96 ± 2.70%. Also, a fat-soluble vitamin E obtained from the roots mainly contains (23.69 ± 2.37%).

**Table 3:** Vitamin contents of *P. Tuberosa* L.

Vitamin content in mg/ 100 g^a^							
*P. tuberosa*	Vitamin C	Vitamin E	Vitamin B1	Vitamin B2	Vitamin B3	Vitamin B5	Vitamin B6
Flowers	21.02 ± 2.10	11.39 ± 1.14	2.14 ± 0.21	1.38 ± 0.14	55.43 ± 5.54	0.69 ± 0.07	2.74 ± 0.27
Roots	26.96 ± 2.70	23.69 ± 2.37	1.02 ± 0.10	0.85 ± 0.09	9.99 ± 1.00	1.46 ± 0.15	0.53 ± 0.05

^a^Values expressed are means ± S.E.M. of three parallel measurements (*p* < 0.05).

### Antioxidant activity

All of the samples exhibited antioxidant activity in a dose-dependent manner. Table 4 shows the IC_50_ antioxidant activity values of essential oils, extracts and standard compounds (α-tocopherol, BHA and EDTA). In all five methods we used to determine antioxidant activity, the methanol extract of three different parts (flower, aerial part and underground) of *P. tuberosa* exhibited higher activity than the hexane extract. In the analyses performed with β-carotene–linoleic acid, ABTS^+^ and CUPRAC methods, the methanol extract of the flowers (IC_50_: 48.35 ± 0.84, 51.93 ± 0.85 and 65.43 ± 0.27 µg/mL, respectively) exhibited higher activity than all other extracts or essential oils. The methanol extract of the aerial part of *P. tuberosa* showed the highest activity in DPPH• free radical scavenging (IC_50_: 66.47 ± 0.28 µg/mL) and iron ion chelation (IC_50_: 122.8 ± 1.17 µg/mL) (Table 4). According to the reducing power capacity (CUPRAC) method, the methanol extract of *P. tuberosa* flower and aerial parts (A_0.50_: 65.43 ± 0.27 and 68.51 ± 0.28 µg/mL, respectively) is competes with the standard antioxidant α-tocopherol (A_0.50_:60.45 ± 0.30 µg/mL). Essential oils obtained from *P. tuberosa* flowers and aerial parts showed higher inhibition in the β-carotene–linoleic acid method (IC_50_: 171.4 ± 1.07 and 194.7 ± 0.89 µg/mL, respectively) compared to other methods. On the other hand, in the other methods, IC_50_ values were determined to be higher than 200 µg/mL.

**Table 4:** Antioxidant activity of the samples by β-Carotene-linoleic acid, DPPH^•^, ABTS^•+^, CUPRAC and metal chelating assays^a^.

Parts of *P*. tuberosa	Samples/ Standards	β-Carotene-linoleic acid assay		DPPH^•^ assay		ABTS^•+^ assay		CUPRAC assay		Metal chelating assay	
		IC_50_ (µg/mL)	Inhibition (%) (at 200 µg/mL)	IC_50_ (µg/mL)	Inhibition (%) (at 200 µg/mL)	IC_50_ (µg/mL)	Inhibition (%) (at 200 µg/mL)	A_0.50_ (µg/mL)	Absorbance (at 200 µg/mL)	IC_50_ (µg/mL)	Inhibition (%) (at 200 µg/mL)
Flowers	Hexane	> 200	27.81 ± 0.66	> 200	17.15 ± 0.20	> 200	19.81 ± 0.51	> 200	0.38 ± 0.01	> 200	35.54 ± 0.76
	Methanol	48.35 ± 0.84	77.20 ± 0.95	73.56 ± 0.51	72.08 ± 0.48	51.93 ± 0.85	76.85 ± 0.65	65.43 ± 0.27	0.86 ± 0.08	158.3 ± 0.97	53.44 ± 1.12
Aerial parts	Hexane	> 200	40.12 ± 0.51	> 200	24.57 ± 0.34	> 200	37.57 ± 0.60	> 200	0.44 ± 0.05	> 200	47.80 ± 0.88
	Methanol	61.12 ± 0.50	75.39 ± 0.46	66.47 ± 0.28	74.58 ± 0.90	52.80 ± 0.39	77.04 ± 0.75	68.51 ± 0.28	0.84 ± 0.07	122.8 ± 1.17	59.10 ± 0.70
Roots	Hexane	> 200	20.27 ± 0.18	> 200	16.29 ± 0.23	> 200	27.71 ± 0.34	> 200	0.33 ± 0.02	> 200	33.20 ± 0.72
	Methanol	85.47 ± 0.94	64.14 ± 0.69	123.9 ± 0.55	58.70 ± 0.84	77.37 ± 1.05	73.10 ± 0.46	95.78 ± 0.35	0.62 ± 0.04	195.1 ± 1.25	51.27 ± 1.08
**Flowers**	Essential oil	171.4 ± 1,07	5129 ± 0.32	> 200	37.23 ± 0.36	> 200	39.23 ± 0.74	> 200	0.43 ± 0.08	> 200	43.15 ± 0.97
Aerial parts	Essential oil	194.7 ± 0,89	50.03 ± 0.64	> 200	34.73 ± 0.58	> 200	41,17 ± 0.88	> 200	0.45 ± 0.12	> 200	42.67 ± 0.53
α-Tocopherol		2.10 ± 0.05	95.73 ± 0.44	38.20 ± 0.50	84.25 ± 0.36	34.75 ± 0.55	83.63 ± 0.34	60.45 ± 0.30	0.88 ± 0.03	NT	NT
BHA		1.50 ± 0.03	96.20 ± 0.28	19.50 ± 0.30	87.90 ± 0.44	12.70 ± 0.10	88.93 ± 0.21	25.40 ± 0.42	2.45 ± 0.07	NT	NT
EDTA		NT	NT	NT	NT	NT	NT	NT	NT	5.50 ± 0.25	94.40 ± 0.35

^a^ Values represent the means ± SEM of three parallel sample measurements (*p* < 0.05). NT: not tested.

### Anticholinesterase activity

In this study we investigated the anticholinesterase enzyme inhibition activity of extracts and essential oil from various parts of *P. tuberosa*. The results are given in Table 5. The inhibitory activities of the extracts on AChE and BChE were reported to be strong (> 50%), moderate (30–50%), inactive or low (< 30%) activity^35^. According to this classification, the essential oil of the flowers of *P. tuberosa* showed strong inhibitory activity against BChE (IC_50_: 194.2 ± 1.05 µg/mL), while the essential oil of the aerial parts (42.65%), flowers (35.97 and 47.31%, respectively), hexane and methanol extracts of the aerial parts (31.70 and 44.60%) showed moderate inhibitory activity. Both hexane (39.67%) and methanol (31.43%) extracts of root parts showed moderate inhibitory activity against AChE (Table 5).

**Table 5:** Antidiabetic, cholinesterase, urease and tyrosinase inhibitory activities of the samples^a^.

Parts of *P*. tuberosa	Extracts/ Standards	Cholinesterase inhibitory activity				Anti-diabetic activity				Urease inhibitory		Tyrosinase inhibitory	
		AChE		BChE		α-glucosidase		α-amylase					
		IC_50_ (µg/mL)	Inhibition (%) (at 200 µg/mL)	IC_50_ (µg/mL)	Inhibition (%) (at 200 µg/mL)	IC_50_ (µg/mL)	Inhibition (%) (at 200 µg/mL)	IC_50_ (µg/mL)	Inhibition (%) (at 200 µg/mL)	IC_50_ (µg/mL)	Inhibition (%) (at 200 µg/mL)	IC_50_ (µg/mL)	Inhibition (%) (at 200 µg/mL)
Flowers	Hexane	> 200	29.53 ± 0.12	> 200	35.97 ± 0.87	> 200	41.77 ± 0.74	152.2 ± 0.46	52.95 ± 1.05	> 200	11.53 ± 0.38	> 200	23.43 ± 0.44
	Methanol	> 200	33.19 ± 0.18	> 200	47.31 ± 0.88	> 200	12.21 ± 0.33	> 200	17.45 ± 0.30	> 200	20.69 ± 0.47	> 200	31.97 ± 1.07
Aerial parts	Hexane	> 200	23.40 ± 0.45	> 200	31.70 ± 0.33	> 200	24.19 ± 0.11	161.6 ± 0.92	52.10 ± 0.96	> 200	15.40 ± 0.35	> 200	19.20 ± 0.14
	Methanol	> 200	29.67 ± 0.22	> 200	44.60 ± 0.10	> 200	10.05 ± 0.22	> 200	20.03 ± 0.75	> 200	22.88 ± 0.64	> 200	30.78 ± 0.51
Roots	Hexane	> 200	39.67 ± 0.92	> 200	25.44 ± 0.23	103.2 ± 0.99	61.36 ± 0.77	90.27 ± 0.25	63.54 ± 0.46	> 200	12.92 ± 0.27	> 200	14.81 ± 0.36
	Methanol	> 200	31.43 ± 0.34	> 200	21.41 ± 0.15	> 200	18.23 ± 0.11	> 200	15.12 ± 0.11	> 200	19.50 ± 0.61	> 200	21.47 ± 0.83
Flowers	Essential oil	> 200	42.16 ± 0.38	194.2 ± 1.05	50.02 ± 0.63	> 200	38.91 ± 0.51	168.3 ± 1.07	51.67 ± 1.19	> 200	27.43 ± 0.19	> 200	31.58 ± 0.27
Aerial parts	Essential oil	> 200	31.67 ± 0.55	> 200	42.65 ± 0.46	> 200	32.12 ± 0.35	185.3 ± 0.92	50.93 ± 0.81	> 200	29.52 ± 0.71	> 200	29.34 ± 0.51
Standards	Galantamine	5.50 ± 0.20	89.25 ± 0.48	42.20 ± 0.35	79.43 ± 0.60	*NT*	*NT*	*NT*	*NT*	*NT*	*NT*	*NT*	*NT*
	Acarbose	*NT*	*NT*	*NT*	*NT*	128.5 ± 0.62	56.70 ± 0.75	32.50 ± 0.45	82.10 ± 0.27	*NT*	*NT*	*NT*	*NT*
	Thiourea	*NT*	*NT*	*NT*	*NT*	*NT*	*NT*	*NT*	*NT*	8.20 ± 0.36	87.37 ± 0.52	*NT*	*NT*
	Kojic acid	*NT*	*NT*	*NT*	*NT*	*NT*	*NT*	*NT*	*NT*	*NT*	*NT*	23.50 ± 0.44	83.54 ± 0.56

^a^ Values represent the means ± SEM of three parallel sample measurements (*p* < 0.05). NT: not tested.

### Anti-tyrosinase activity

The tyrosinase enzyme inhibition activity results of the extracts and essential oil are given in Table 5. As shown in Table 5, methanol extracts (31.97 ± 1.07%, 30.78 ± 0.51% and 21.47 ± 0.83%) of *P. tuberosa* flowers, above-ground and root parts exhibited higher activity compared to hexane extracts (23.43 ± 0.44%, 19.20 ± 0.14% and 14.81 ± 0.36%) but lower activity compared to the reference compound kojic acid (83.54 ± 0.56%). In addition, the essential oil of flowers (31.58 ± 0.27%) exhibited higher tyrosinase enzyme inhibition activity than the essential oil of above-ground parts (29.34 ± 0.51%), although both results were lower than the activity of kojic acid.

### Antidiabetic activity

The antidiabetic activities of the extracts and essential oil were determined using α-glucosidase and α-amylase enzymes. The results are presented in Table 5. The hexane extract of the underground part of *P. tuberosa* exhibited an α-glucosidase inhibitory activity (IC_50_:128.5 ± 0.62 µg/mL) exceeding that of acarbose (IC_50_:103.2 ± 0.99 µg/mL). The α-glucosidase enzyme inhibition activity of the other extracts and essential oils we studied was calculated to be at concentrations higher than 200 µg/mL. In addition, a significant α-amylase inhibition activity of essential oils and hexane extracts of various parts of *P. tuberosa* was calculated. Although the hexane extract of the subsoil fraction exhibited higher α-amylase inhibition activity (IC_50_: 90.27 ± 0.25 µg/mL) than the other extracts, it exhibited a lower effect than acarbose.

### Anti-urease activity

It can be seen in Table 5 that the essential oils of the aerial parts and flowers of *P. tuberosa* showed higher urease inhibition activity than all other extracts (29.52 ± 0.71% and 27.43 ± 0.19%, respectively), followed by the methanol extract of the aerial parts. The urease enzyme inhibition activities of all extracts and essential oils showed a low activity compared to the reference substance thiourea (87.37 ± 0.52%), which is a urease inhibitor. According to our results, we determined that all the samples we studied had urease enzyme inhibition activity more than 200 µg/mL (Table 5).

## Discussion

It is understood that previous studies have investigated the chemical components of essential oils obtained from the aerial parts of *P. tuberosa *grown in different places^4,8,12^. Therefore, the chemical components of the essential oil of its flowers were determined for the first time in this research. Differences in the chemical content of essential oils are a direct result of the geographical location where the plant grows (longitude, altitude, and climate), soil composition, and intra-population cross-pollination. It has also been suggested that various variations such as the technique used in obtaining the essential oil, the phenological development stage of the plants, and the season affect the components of the oil^4,11,36^. Amor et al. (2009) suggested that the essential oils of *Phlomis *species can be divided into four main chemotypes according to their major compounds and compound classes^3^. Accordingly, in the first group, those rich in sesquiterpenes such as germacrene D and caryophyllene^37^in the second group, those rich in mono- and sesquiterpenes such as α-pinene, limonene, linalool, germacrene D and β-caryophyllene^38–40^In the third group, there are those rich in aliphatic compounds, diterpenoid alcohols and higher fatty alcohols, such as hexadecanoic acid, trans-phytol and 9,12,15-octadecatrien-1-ol. In the fourth group, terpenes such as hexadecanoic acid, α-pinene and germacrene D are classified as those rich in fatty acids and aliphatic compounds^11^. In this study, we can say that the essential oil obtained from various parts of *P. tuberosa* growing in Kazakhstan overlaps with the third group in terms of its dominant components. The chemical components of the essential oil we obtained from the aerial parts in this study are in harmony with other previous studies. From the identified compounds, n-octacosane possesses the highest quantity as identified in the flowers (14.34%) and the aerial parts (25.66%) of the plant *P. tuberosa.* The compound n-Octacosane is a saturated straight-chain hydrocarbon that has the potential to display in vitro and in vivo antioxidant properties and could be used in the treatment of bacteria and possess potent antitumor functions^41^. Another vital compound with a higher concentration of essential oil is hexahydro farnesyl acetone, This compound recorded 13.89% in the flowers and 18.75% in the aerial part of the sampled plant. Hexahydro farnesyl acetone is a colorless to light yellow terpenoid known to be a significant constituent of essential oil with anti-inflammatory, antioxidant, and anti-nociceptive activities^42^. It possesses structural properties known to be present in most plants and animals, squalene, a polyunsaturated hydrocarbon, is usually found in large amounts in fish oils and relatively smaller quantities in vegetable oils. The results showed that the flower of the plant *P*,* tuberosa*, contains 6.31% while only 0.87% was recorded in the aerial part, In general, squalene has been known to be an effective compound in traditional medicines for several decades due to its bioactive properties such as antioxidant, anticancer, drug carrier, emollient, skin hydrant, and detoxifier, It is also considered an essential compound with potential in the pharmaceutical and nutraceutical industries^43^.

Phenolic compounds are one of the important classes of compounds responsible for antioxidant activity in plants. Flavonoids have important antioxidant and anti-inflammatory functions being capable of scavenging free radicals, inhibit lipoxygenase and cyclooxygenase, chelating transition metals (iron and copper), and protect^44^. Although no research on the isolation of flavonoids from *P. tuberosa* has been carried out so far, apigenin, luteolin, naringenin, eriodictyol, chryseriol, kaempferol and their glycosides have been isolated in studies on other *Phlomis *species^3,45^. When previous research is examined, although there are detailed studies on the iridoids of *P. tuberosa*, there is very limited research on its flavonoid content. Kondeva-Burdina and colleagues conducted in vitro/in vivo hepatotoxicity and hepatoprotection evaluation of a defatted extract and a phenolic fraction from *P. tuberosa* grown in Bulgaria and investigated the phenolic compound profile of the relevant extract and fraction. In the only previous study on the phenolic compounds of *P. tuberosa*, it was determined that luteolin, quercetin, spiraeoside, apigenin-7-glucoside, luteolin-7-glucuronide and verbascoside were found in the aerial parts of the plant grown in Bulgaria^9^.

When the literature is examined, it is seen that antioxidant activity studies on the *Phlomis* genus are limited. In the study conducted by Couladis et al. (2003), it was reported that *Phlomis fruticosa* and *Phlomis lanata *methanol extract had antioxidant activity and prevented arachidonic acid super oxidation catalyzed by Bleomycin-Fe (II)^13^. It was determined that extracts of *P. maximowiczii *with high radical scavenging effect in vitro conditions had a hepatoprotective effect on acute liver damage caused by carbon tetrachloride in mice^46^. Methanol extracts of aerial parts of *P. stewartii* and *P. anisodonta *have been reported to show good antioxidant activity in restoring clinical parameters tested in cigarette smoke/alloxan-induced animals. In addition, these extracts have also been reported to have good antidiabetic, hepatoprotective and nephroprotective potential in diabetic animal models^9,47–49^. In another study, antineurodegenerative, anti-inflammatory, antimicrobial and antioxidant activities of extracts from *P. fruticosa* and *P. russeliana*were proven^9,50–52^. Free radical scavenging activity has been demonstrated for *P. caucasica* and antioxidant effects for *P. lychnitis*, a traditional herbal tea^14,53^. It has been suggested that the phenolic compounds and some iridoids found in these species may be effective on the antioxidant activity of other *Phlomis* species, especially *P. leucophracta*^54^. The results we obtained in this study agree with the antioxidant activity results obtained so far on *Phlomis* species.

There are many drugs used to treat Alzheimer’s Disease (AD) and these drugs have some side effects. Therefore, it is necessary to obtain a new inhibitor that is less toxic for AD. Currently, there are a limited number of inhibitors (galantamine, tacrine and physostigmine) derived from natural plant sources that reduce the effects of AD^55^. Scientists’ interest in finding new sources of natural drugs for the treatment of AD has been increasing recently. In the literature, especially the anticholinesterase activities of essential oils obtained from some *Phlomis* species have been reported. In these studies, the anticholinesterase activities of *P. kurdica*, *P. armeniaca*, *P. nissolii* and *P. pungens *were investigated and it was revealed that the essential oils showed moderate anticholinesterase activity. In this respect, it was observed that the results were similar to our results^56,57^. Therefore, this is the first study on acetylcholinesterase (AChE) and butyrylcholinesterase (BChE) enzyme inhibition activities of extracts and essential oils obtained from various parts of *P. tuberosa*.

In recent years, tyrosinase inhibitors have gained importance due to the effect of tyrosinase on human melanogenesis and browning of plants and fungi. In the method used, tyrosinase enzyme inhibitor activity is based on the measurement of dopachrome formed in the presence of tyrosinase and the enzyme substrate L-DOPA^58^. To date, there are no studies on the anti-tyrosinase activity of extracts or essential oils from *Phlomis* species. Therefore, this study is the first study on the tyrosinase enzyme inhibition activity of extracts and essential oils obtained from various parts of *P. tuberosa.*

Worldwide, the prevalence of diabetes has nearly doubled since 1980, and diabetes is predicted to be the seventh leading cause of death by 2030. When diabetes is not treated appropriately, diabetes can lead to kidney failure, blindness, and other lower extremity amputations, and finally long-term consequences, significantly affecting the quality of life^59^. Inhibition of α-amylase and α-glucosidase by delaying the digestion of carbohydrates and slowing the rate of glucose absorption is recommended as one of the most important strategies used in the treatment of the disease^60,61^. Several *Phlomis* species, such as *P. aurea*,* P. ocymifolia*, and *P. anisodonta*, are known for their antidiabetic properties. It has been suggested that the activities of these species may be due mainly to their ability to protect the integrity of the liver and pancreas by reducing oxidative stress in diabetes or by stimulating the production of enzymes involved in glucose metabolism^3,48^. Sarkhail et al. (2007) investigated the antihyperglycemic activity of *Phlomis anisodonta* methanolic extract in a streptozocin (STZ)-induced diabetes model in rats. In the study, it was determined that methanol extract relieved oxidative stress through antihyperglycemic effect, improving the plasma ferric-reducing antioxidant power of PAME, reducing liver lipid peroxidation and activation of hepatic antioxidant enzymes. Thus, diabetic rats treated with *P. anisodonta *methanol extract showed a significant increase in hepatic superoxide dismutase, catalase and glutathione peroxidase activities^48^. In vitro antidiabetic activity potentials of various extracts obtained from *P. auera* and *P. monocephala *are available in the literature^62,63^. It was reported that ethyl acetate extract obtained from *P. auera* showed higher activity with IC_50_values of 1.99 mg/mL (α-amylase) and 1.22 mg/mL (α-glucosidase)^62^, while in the other study, dichloromethane extract obtained from *P. monocephala *exhibited potent activity with a value of 2.40 mmol Acarbose/g extract^63^. To date, there have been no studies conducted to date regarding the in vitro antidiabetic activity of *P. tuberosa*. Therefore, this study is the first study on the alpha-glucosidase and alpha-amylase enzyme inhibition activities of extracts and essential oils obtained from various parts of *P. tuberosa*.

*Helicobacter pylori*, which can live in the acidic environment of the stomach, causes health problems such as gastroenteritis, gastric lymphoma, gastric adenocarcinoma, stomach and duodenal ulcers. At this point, it is accepted that the release of ureases, which cause hydrolysis of urea bound to the surface of healthy bacterial cells, increases with the lysis of some pathogenic cells. The use of urease inhibitors in the treatment of urease-related diseases attracts attention as a new treatment method adopted in recent years. However, due to the toxicity of commercially available urease inhibitors and their properties that hinder their clinical use, scientists have turned their research to natural sources^64^. There are no studies conducted to date regarding the urease enzyme inhibition activity of extracts or essential oils obtained from *Phlomis* species. Therefore, this study is the first study on the urease enzyme inhibition activities of extracts and essential oils obtained from various parts of *P. tuberosa*.

## Conclusions

In this study, the antioxidant, enzyme inhibitory properties as well as phenolic compound contents and volatile oil constituents of various extracts and essential oils of the flowers, aerial part and roots of *P. tuberosa*, which is naturally distributed in Ortau Mountains, were investigated. According to β-carotene-linoleic acid, ABTS^+^ and CUPRAC methods, the methanol extract of flowers exhibited higher antioxidant activity than all other extracts. The methanol extract of the aerial part of *P. tuberosa* had the highest activity in DPPH free radical scavenging and iron chelating methods. While the essential oil of *P. tuberosa* flowers showed strong BChE inhibitory activity, hexane and methanol extracts of the aerial parts showed moderate AChE inhibitory activity. Although the urease inhibitory effect of both essential oils was higher than that of the extracts, the urease inhibitory effect of *P. tuberosa* was lower compared to thiourea. The tyrosinase enzyme inhibitory effect of methanol extracts were found to be higher than other extracts. Essential oils and hexane extracts showed significant α-amylase inhibition activity. The hexane extract from *P. tuberosa* roots has higher a-glucosidase inhibitory activity than acarbose. A total of 10 phenolic compounds were detected in methanol extracts of different parts of the plant using HPLC-DAD. Ferulic acid and chlorogenic acid are the common main compounds in three different parts of *P. tuberosa*. The flowers and aerial parts are also rich in rosmarinic acid, coumarin and luteolin. Significant amounts of vitamins B1, B2, B3, B5 and B6 as well as significant amounts of vitamin C and E were detected in *P. tuberosa* roots. The chemical composition of the essential oils obtained from the flowers and aerial parts were analyzed using GC and GC-MS systems and a total of 51 components were determined. Both essential oils are richer in hydrocarbons and sesquiterpenoids. The essential oil constituents of flowers and aerial parts do not differ much qualitatively, but quantitatively they differ significantly. We believe that the presence of hexahydro farnesyl acetone and squalene, which have a wide range of bioactivities, in significant amounts in both essential oils will improve the quality of essential oils in terms of their biological activities. This study represents the first report on the detailed analysis of various parts of *P. tuberosa* for their phenolic compounds, vitamins, in vitro anti-diabetic, anti-cholinesterase, urease and tyrosinase enzyme inhibitory activities. Our results showed that this species has promising bioactivities and high phytochemical content. They can be used as a natural source of Vitamin C, Vitamin E and Vitamin B3, antioxidant, antidiabetic, anticholinesterase and anti-tyrosinase agent in food, cosmetic and pharmaceutical industries. Therefore, further studies are needed to isolate and identify antioxidant and antidiabetic components from these species.

## Data Availability

The datasets used and/or analysed during the current study available from the corresponding author on reasonable request.

## References

[1] Kinghorn, A. D., Pan, L., Fletcher, J. N. & Chai, H. The relevance of higher plants in lead compound discovery programs. *J. Nat. Prod.* **74**(6), 1539–1555 (2011). 21650152 10.1021/np200391c PMC3158731

[2] Chen, J., Li, W., Yao, H. & Xu, J. Insights into drug discovery from natural products through structural modification. *Fitoterapia* **103**, 231–241 (2015). 25917513 10.1016/j.fitote.2015.04.012

[3] Amor, I. L. B. et al. Chekir-Ghedira, L. Phytochemistry and biological activities of Phlomis species. *J. Ethnopharmacol.* **125**, 183–202 (2009). 19563875 10.1016/j.jep.2009.06.022

[4] Vitaliy, K. et al. Chemical composition of essential oil from Aerial Parts of *Phlomis tuberosa* L. growing Wild in Northern Kazakhstan. *J. Essent. Oil-Bear Plants*. **21**(2), 462–475 (2018).

[5] Alipieva, K. I., Jensen, S. R., Franzyk, H., Handjieva, N. V. & Evstatieva, L. N. Iridoid Glucosides from *Phlomis tuberosa* L. and *Phlomis herba-ventis* L. *Z. Naturforsch C J. Biosci.* **55**(3–4), 137–140 (2000). 10817200 10.1515/znc-2000-3-402

[6] Yang, Y. et al. Rapid Identification of α-Glucosidase inhibitors from *Phlomis tuberosa* by Sepbox Chromatography and Thin-Layer Chromatography Bioautography. *PLoS One* **10**(2), e0116922 (2015). 10.1371/journal.pone.0116922 PMC4319760 25658100

[7] Olennikov, D. N. & Chirikova, N. K. Phlotuberosides I and II, New Iridoid glycosides from *Phlomoides tuberosa*. *Chem. Nat. Compd.* **53**, 269–272 (2017).

[8] Javzan, S. & Selenge, D. Phytochemical study of aerial parts from *Phlomis tuberosa* L. *Mong J. Chem.* **14**, 20–24 (2014).

[9] Kondeva-Burdina, M., Shkondrov, A., Popov, G., Manov, V. & Krasteva, I. In Vitro/In vivo hepatoprotective and antioxidant effects of Defatted Extract and Phenolic Fraction obtained from *Phlomis Tuberosa*. *Int. J. Mol. Sci.* **24**, 10631 (2023). 37445808 10.3390/ijms241310631 PMC10341447

[10] Guven, L., Erturk, A., Koca, M. & Gulcin, I. Phenolic Compounds of *Phlomis tuberosa* by LC–MS/MS-Determination of Antioxidant Activity, Molecular Docking, and Enzyme Inhibition Profiles. *ChemistrySelect* **8**, e202303101 (2023).

[11] Zhang, Y. & Wang, Z. Z. Comparative analysis of essential oil components of three Phlomis species in Qinling Mountains of China. *J. Pharma Biomed. Anal.* **47**(1), 213–217 (2008). 10.1016/j.jpba.2007.12.027 18243625

[12] Olennikov, D. N., Dudareva, L. V. & Tankhaeva, L. M. Chemical composition of essential oils from *Galeopsis bifida* and *Phlomoides tuberosa*. *Chem. Nat. Compd.* **46**, 316–318 (2010).

[13] Couladis, M., Tzakou, O., Verykokidou, E. & Harvala, C. Screening of some Greek aromatic plants for antioxidant activity. *Phytother Res.* **17**, 194–195 (2003). 12601688 10.1002/ptr.1261

[14] Delazar, A. et al. Free-radical-scavenging principles from *Phlomis Caucasica*. *J. Nat. Med.* **62**, 464–466 (2008). 18484154 10.1007/s11418-008-0255-y

[15] Coelho, S. C., Estevinho, B. N. & Rocha, F. Recent advances in Water-Soluble vitamins Delivery systems prepared by mechanical processes (Electrospinning and Spray-Drying techniques) for food and Nutraceuticals Applications—A Review. *Foods* **11**(9), 1271 (2022). 35563994 10.3390/foods11091271 PMC9100492

[16] Djebili, S. et al. Volatile compound profile and essential oil composition of three wild Algerian aromatic plants with their antioxidant and antibiofilm activities. *J. Food Meas. Charact.* **16**, 987–999 (2022).

[17] Adams, R. P. *Identification of Essential oil Components by gas chromatography/mass Spectrometry* 4th edn (Allured Publishing Corporation, 2007).

[18] ISC, Interstate Council For Standardization, Metrology And Certification (ISC). (2022).

[19] ROSSTANDART, Federal Agency For Technical Regulation And Metrology. (2023). http://government.ru/en/department/56/

[20] Duru, M. E. et al. HPLC‐DAD analysis and versatile bioactivities of Turkish sunflower honeys using chemometric approaches. *Chem. Biodivers.* **20**, 6, e202300486 (2023). 37192321 10.1002/cbdv.202300486

[21] Marco, G. J. A Rapid Method for Evaluation of Antioxidants. *J. Am. Oil Chem. Soc.* **45**, 594–598 (1968).

[22] Tel-Çayan, G. & Duru, M. E. Chemical characterization and antioxidant activity of *Eryngium pseudothoriifolium* and *E. Thorifolium* essential oils. *J. Res. Pharm.* **23** (2019).

[23] Çayan, F. et al. Application of GC, GC-MSD, ICP-MS and spectrophotometric methods for the determination of Chemical Composition and in Vitro Bioactivities of *Chroogomphus rutilus*: the Edible Mushroom species. *Food Anal. Methods*. **7**, 449–458 (2014).

[24] Re, R. et al. Antioxidant activity applying an Improved ABTS Radical Cation Decolorization Assay. *Free Radic Biol. Med.* **26**, 1231–1237 (1999). 10381194 10.1016/s0891-5849(98)00315-3

[25] Apak, R., Güçlü, K., Özyürek, M. & Karademir, S. E. Novel total antioxidant Capacity Index for Dietary polyphenols and vitamins C and E, using their Cupric Ion reducing capability in the Presence of Neocuproine: CUPRAC Method. *J. Agric. Food Chem.* **52**, 7970–7981 (2004). 15612784 10.1021/jf048741x

[26] Tel, G., Apaydın, M., Duru, M. E. & Öztürk, M. Antioxidant and Cholinesterase Inhibition Activities of Three *Tricholoma* Species with total phenolic and flavonoid contents: the Edible mushrooms from Anatolia. *Food Anal. Methods*. **5**, 495–504 (2012).

[27] Deveci, E., Tel-Çayan, G., Usluer, Ö. & Duru, M. E. Chemical composition, antioxidant, Anticholinesterase and anti-tyrosinase activities of essential oils of two *Sideritis* species from Turkey. *Iran. J. Pharm. Res.* **18**(2), 903–913 (2019). 31531072 10.22037/ijpr.2019.1100657 PMC6706731

[28] Tamfu, A. N., Kucukaydin, S., Ceylan, O., Sarac, N. & Duru, M. E. Phenolic composition, enzyme inhibitory and anti-quorum sensing activities of Cinnamon (*Cinnamomum zeylanicum* Blume) and Basil (*Ocimum basilicum* Linn). *Chem. Afr.* **4**, 759–767 (2021).

[29] Masuda, T., Yamashita, D., Takeda, Y. & Yonemori, S. Screening for tyrosinase inhibitors among extracts of seashore plants and identification of potent inhibitors from *Garcinia Subelliptica*. *Biosci. Biotechnol. Biochem.* **69**, 197–201 (2005). 15665485 10.1271/bbb.69.197

[30] Küçükaydın, S., Çayan, F., Tel-Çayan, G. & Duru, M. E. HPLC-DAD phytochemical profiles of *Thymus cariensis* and *T. cilicicus* with antioxidant, cytotoxic, anticholinesterase, anti-urease, anti-tyrosinase, and antidiabetic activities. *S Afr. J. Bot.* **143**, 155–163 (2021).

[31] Kim, J. S., Kwon, C. S. & Son, K. H. Inhibition of α-glucosidase and amylaze by luteolin, a flavonoid. *Biosci. Biotechnol. Biochem.* **64**, 2458–2461 (2010). 10.1271/bbb.64.2458 11193416

[32] Alum, E. U., Aja, W., Ugwu, O. P. C. & Obeagu, E. I. Ben Okon, M. Assessment of Vitamin Composition of Ethanol Leaf and seed extracts of *Datura Stramonium*. *Avicenna J. Med. Biochem.* **11**(1), 92–97 (2023).

[33] Webb, M. E. & Smith, A. G. Pantothenate biosynthesis in higher plants. *Adv. Bot. Res.* **58**, 203–255 (2011).

[34] Igile, G. O., Iwara, I. A., Mgbeje, B. I. A., Uboh, F. E. & Ebong, P. E. Phytochemical, Proximate and Nutrient Composition of *Vernonia calvaona* Hook (Asterecea): a Green-Leafy Vegetable in Nigeria. *J. Food Res.* **2**(6), 1–11 (2013).

[35] Vinutha, B. et al. Screening of selected Indian Medicinal plants for acetylcholinesterase inhibitory activity. *J. Ethnopharmacol.* **109**, 359–363 (2007). 16950584 10.1016/j.jep.2006.06.014

[36] Duru, M. E., Cakir, A. & Harmandar, M. Composition of the volatile oils isolated from the leaves of *Liquidambar orientalis* Mill. var. *orientalis* and *L. orientalis* var. *integriloba* from Turkey. *Flavour Fragr. J*. **17**(2), 95–98 (2002).

[37] Basta, A., Tzakou, O. & Couladis, M. The essential oil composition of *Phlomis Cretica* C. Presl. *Flavour. Fragr. J.* **21**(5), 795–797 (2006).

[38] Aligiannis, N., Kalpoutzakis, E., Kyriakopoulou, I., Mitaku, S. & Chinou, I. Essential oils of *Phlomis* species growing in Greece: Chemical composition and antimicrobial activity. *Flavour. Fragr. J.* **19**(4), 320–324 (2004).

[39] Celik, S., Gokturk, R., Flamini, G., Cioni, P. & Morelli, I. Essential oils of Phlomis Leucophracta, Phlomis chimerae and Phlomis Grandiflora var. Grandiflora from Turkey. *Biochem. Syst. Ecol.* **33**(6), 617–623 (2005).

[40] Liolios, C., Laouer, H., Boulaacheb, N., Gortzi, O. & Chinou, I. Chemical composition and antimicrobial activity of the essential oil of Algerian Phlomis bovei subsp. bovei. *Molecules* **12**(4), 772–781. 10.3390/12040772 (2007). 17851429 10.3390/12040772 PMC6149321

[41] Bouazzi, S. et al. Chemical composition and antioxidant activity of essential oils and hexane extract of *Onopordum arenarium* from Tunisia. *J. Chromatogr. Sci.* **58**(4), 287–293 (2020). 31867630 10.1093/chromsci/bmz113

[42] Avoseh, O. N., Mtunzi, F. M., Ogunwande, I. A., Ascrizzi, R. & Guido, F. *Albizia lebbeck* and *Albizia zygia* volatile oils exhibit anti-nociceptive and anti-inflammatory properties in pain models. *J. Ethnopharmacol.* **268**, 113676 (2021). 33301915 10.1016/j.jep.2020.113676

[43] Kim, S. K. & Karadeniz, F. Biological Importance and Applications of Squalene and Squalane. *Adv. Food Nutr. Res.* **65**, 223–233 (2012). 22361190 10.1016/B978-0-12-416003-3.00014-7

[44] Kahraman, A., Serteser, M., Koken, T. & Flavonoids *Med. J. Kocatepe* **3**(1), 1–8 (2002).

[45] Marin, P. D. et al. Flavonoids from *Phlomis fruticosa* (Lamiaceae) growing in Montenegro. *Biochem. Syst. Ecol.* **35**, 462–466 (2007).

[46] Gu, H., Gu, X., Xu, Q. & Kang, W. Antioxidant activity in Vitro and Hepatoprotective Effect of *Phlomis Maximowiczii* in vivo. *Afr. J. Tradit Complement. Altern. Med.* **11**, 46–52 (2014). 25371563 10.4314/ajtcam.v11i3.8 PMC4202419

[47] Sarkhail, P. et al. Effect of *Phlomis Persica* on glucose levels and hepatic enzymatic antioxidants in Streptozotocin-Induced Diabetic rats. *Pharmacogn Mag*. **6**(23), 219–224 (2010). 20931083 10.4103/0973-1296.66940 PMC2950386

[48] Sarkhail, P. et al. Antidiabetic effect of *Phlomis anisodonta*: effects on hepatic cells lipid peroxidation and antioxidant enzymes in experimental diabetes. *Pharmacol. Res.* **56**, 261–266 (2007). 17714953 10.1016/j.phrs.2007.07.003

[49] Rasheed, M. U., Naqvi, S. A. R., Rasool, N., Shah, S. A. A. & Zakaria, Z. A. Anti-diabetic and cytotoxic evaluation of *Phlomis stewartii* plant phytochemicals on cigarette smoke inhalation and Alloxan-Induced diabetes in Wistar rats. *Metabolites* **12**, 1133 (2022). 36422273 10.3390/metabo12111133 PMC9696311

[50] Ferrante, C. et al. Protective effects Induced by Alcoholic *Phlomis fruticosa* and *Phlomis herba-venti* extracts in isolated rat Colon: focus on Antioxidant, anti-inflammatory, and Antimicrobial activities in Vitro. *Phytother Res.* **33**, 2387–2400 (2019). 31322313 10.1002/ptr.6429

[51] Stojković, D. et al. Chemical profiling, antimicrobial, anti-enzymatic, and cytotoxic properties of *Phlomis fruticosa* L. *J. Pharm. Biomed. Anal.* **195**, 113884 (2021). 33421668 10.1016/j.jpba.2020.113884

[52] Stojkovic, D. et al. *Phlomis fruticosa* L. exerts in vitro antineurodegenerative and antioxidant activities and induces prooxidant effect in glioblastoma cell line. *EXCLI J.* **21**, 387–399 (2022). 35368464 10.17179/excli2021-4487 PMC8971322

[53] López, V., Jäger, A. K., Akerreta, S., Cavero, R. Y. & Calvo, M. I. Antioxidant activity and phenylpropanoids of *Phlomis Lychnitis* L.: a traditional herbal tea. *Plant. Foods Hum. Nutr.* **65**, 179–185 (2010). 20422294 10.1007/s11130-010-0169-1

[54] Sarikurkcu, C. & Ćavar Zeljković, S. Chemical composition and antioxidant activity of *Phlomis Leucophracta*, an endemic species from Turkey. *Nat. Prod. Res.* **34**(6), 851–854 (2020). 30417666 10.1080/14786419.2018.1502767

[55] Salah, S. M. & Jäger, A. K. Screening of traditionally used Lebanese herbs for neurological activities. *J. Ethnopharmacol.* **97**, 145–149 (2005). 15652288 10.1016/j.jep.2004.10.023

[56] Karadağ, A. E., Demirci, B., Kültür, Ş., Demirci, F. & Başer, K. H. C. Antimicrobial, anticholinesterase evaluation and chemical characterization ofessential oil *Phlomis Kurdica* Rech. Fil. Growing in Turkey. *J. Essent. Oil Res.* **32**(3), 242–246 (2020).

[57] Sarikurkcu, C., Uren, M. C., Kocak, M. S., Cengiz, M. & Tepe, B. Chemical composition, antioxidant, and enzyme inhibitory activities of the essential oils of three *Phlomis* species as well as their fatty acid compositions. *Food Sci. Biotechnol.* **23**(3), 687–693 (2016). 10.1007/s10068-016-0120-9 PMC6049173 30263324

[58] Chang, T. S. An updated review of tyrosinase inhibitors. *Int. J. Mol. Sci.* **10**, 2440–2475 (2009). 19582213 10.3390/ijms10062440 PMC2705500

[59] Salehi, B. et al. W. C. & Sharifi-Rad, J. Antidiabetic Potential of Medicinal Plants and Their Active Components. *Biomolecules* **9**(10), 551 (2019). 10.3390/biom9100551 PMC6843349 31575072

[60] Raman, B. V., Krishna, A. N. V., Rao, B. N., Saradhi, M. P. & Rao, M. V. B. plants with antidiabetic activities and their medicinal values. *Int. Res. J. Pharm.* **3**(3), 11–15 (2012).

[61] Tamfu, A. N. et al. Phenolic composition, antioxidant and enzyme inhibitory activities of *Parkia biglobosa* (Jacq.) Benth., *Tithonia diversifolia* (Hemsl) A. Gray, and *Crossopteryx Febrifuga* (Afzel.) Benth. *Arab. J. Chem.* **15**(4), 103675 (2022).

[62] El-Azab, M. M., Ibrahim, M. A., El-Bassossy, T. A. I. & Ahmed, F. A. In vitro anti-diabetic effect and molecular docking study of *Phlomis aurea* components as diabetic enzymes inhibitor. *Egypt. J. Chem.* **67**(10), 209–224 (2024).

[63] Zheleva-Dimitrova, D. et al. Deciphering the chemical constituents of *Phlomis monocephala* extracts using UHPLC-HRMS and their antioxidant, neuroprotective, antidiabetic and toxic potentials. *Food Biosci.* **59**, 104183 (2024).

[64] Modolo, L. V., de Souza, A. X., Horta, L. P., Araujo, D. P. & Fátima A. P. An overview on the potential of natural products as ureases inhibitors: a review. *J. Adv. Res.* **6**, 35–44 (2015). 25685542 10.1016/j.jare.2014.09.001 PMC4293669

